# A machine learning approach to estimation of downward solar radiation from satellite-derived data products: An application over a semi-arid ecosystem in the U.S.

**DOI:** 10.1371/journal.pone.0180239

**Published:** 2017-08-04

**Authors:** Qingtao Zhou, Alejandro Flores, Nancy F. Glenn, Reggie Walters, Bangshuai Han

**Affiliations:** 1 Department of Geosciences, Boise State University, Boise, Idaho, United States of America; 2 Department of Natural Resources and Environmental Management, Muncie, Indiana, United States of America; Tennessee State University, UNITED STATES

## Abstract

Shortwave solar radiation is an important component of the surface energy balance and provides the principal source of energy for terrestrial ecosystems. This paper presents a machine learning approach in the form of a random forest (RF) model for estimating daily downward solar radiation flux at the land surface over complex terrain using MODIS (MODerate Resolution Imaging Spectroradiometer) remote sensing data. The model-building technique makes use of a unique network of 16 solar flux measurements in the semi-arid Reynolds Creek Experimental Watershed and Critical Zone Observatory, in southwest Idaho, USA. Based on a composite RF model built on daily observations from all 16 sites in the watershed, the model simulation of downward solar radiation matches well with the observation data (r^2^ = 0.96). To evaluate model performance, RF models were built from 12 of 16 sites selected at random and validated against the observations at the remaining four sites. Overall root mean square errors (RMSE), bias, and mean absolute error (MAE) are small (range: 37.17 W/m^2^-81.27 W/m^2^, -48.31 W/m^2^-15.67 W/m^2^, and 26.56 W/m^2^-63.77 W/m^2^, respectively). When extrapolated to the entire watershed, spatiotemporal patterns of solar flux are largely consistent with expected trends in this watershed. We also explored significant predictors of downward solar flux in order to reveal important properties and processes controlling downward solar radiation. Based on the composite RF model built on all 16 sites, the three most important predictors to estimate downward solar radiation include the black sky albedo (BSA) near infrared band (0.858 μm), BSA visible band (0.3–0.7 μm), and clear day coverage. This study has important implications for improving the ability to derive downward solar radiation through a fusion of multiple remote sensing datasets and can potentially capture spatiotemporally varying trends in solar radiation that is useful for land surface hydrologic and terrestrial ecosystem modeling.

## 1. Introduction

### 1.1. Background

Shortwave (0.3–5.0 μm) solar radiation is the principal source of energy to drive photosynthesis in Earth’s terrestrial ecosystems. As such, characterizing the measurement and spatiotemporal variation in solar fluxes is important in physics, biology, chemistry, hydrology, and other natural sciences. Additionally, solar radiation is the largest component of the available energy to drive evaporation from the surface, underscoring its importance as a variable that connects land-atmosphere fluxes. Because of its role in controlling surface energy balance, moreover, solar radiation indirectly contributes to soil microbial processes through its impact on ground heat flux and the subsurface distribution and dynamics of soil temperature. Changes in solar radiation are associated with global biogeochemical cycling through impacts on photosynthesis. Analyses of tropical Net Primary Production (NPP), for instance, suggest that increasing solar radiation has led to increases in NPP [1]. Evapotranspiration (ET) is dependent on downward solar radiation, which provides the energy to evaporate water. Based on previous studies [2, 3], both pan evaporation and downward solar radiation have decreased over the last 50 years.

Downward solar radiation flux is also an important land surface parameter for ecological, land surface hydrology, and weather forecast models such as the Community Land Model [4], Biome-biogeochemical (Biome-BGC) [5], Photosynthesis evapotranspiration—biogeochemical model (PnET-BGC) [6–8], general circulation models (GCMs) [9] and the Weather Research and Forecasting Model (WRF) [10]. Within these models the downward solar radiation flux is either required as an input parameter or, in the case of WRF and other coupled land-atmosphere models, produced as an output parameter. The accuracy of the input downward solar radiation directly affects the corresponding accuracy of model outputs related to surface energy budgets like primary production, evapotranspiration, and indirectly impacts other parameters such as infiltration, runoff, and chemical solutions in the stream water [9, 11–12]. Models of coupled land-atmosphere dynamics, such as WRF, produce solar radiation fluxes as an output, capturing the impact of clouds on surface solar radiation either through parameterizations or by explicitly resolving clouds. The ability to verify model-predicted solar radiation at the surface against observational information, therefore, would enhance the ability to assess and characterize errors of both land surface hydrological states and fluxes and also the effects of simulated atmospheric conditions on the attenuation of solar radiation from the top of the atmosphere. Observational information used for verification of input or output surface downwelling solar flux would ideally capture spatiotemporal patterns in solar radiation at spatial resolutions approaching those of the model being used. However, most observational solar flux information is available only at the point scale. The ability to deduce spatial correlates of solar radiation from networks of point-based surface observations and use that information to generate spatiotemporal predictions of downward solar flux would, therefore, substantially improve land modeling efforts.

Traditionally, three different methods have been used for obtaining downward solar radiation information, all of which have strengths and limitations. Ground-based pyranometers are a relatively inexpensive way to obtain estimates of hemispherical solar radiation flux that use a voltage-generating thermopile that is excited by exposure to solar radiation. While they provide accurate estimates of solar radiation with high temporal resolution, networks of pyranometers are typically not available in sufficiently high spatial coverage to resolve spatial patterns [13]. Sparseness in spatial coverage is particularly prevalent in complex and mountainous terrain where placing monitoring stations is logistically challenging. An alternative method for calculating solar radiation is to use mathematical or empirical models. The method of Hargreaves and Samani [14] uses maximum and minimum daily temperature to estimate the downward solar radiation. Although this empirical method for estimating solar radiation is relatively simple and can be made with commonly available meteorological observations, it is based on the assumption that solar radiation is related to the difference between maximum and minimum temperature and the fraction of extraterrestrial radiation received at the ground level, which results in model uncertainties. Other models such as the Angstrom-Prescott model [15–16] use site-specific model parameters to obtain downward solar radiation. However, these parameters are based on ground based measurements and limited by these measurements [17]. Finally, a number of studies have used remote sensing data to estimate downward solar radiation using the split window technique [18–19] or look up table method [20]. The major advantage of using remote sensing information is that it provides spatiotemporal coverage of the land surface, which potentially supports the development of long-term databases of downward solar radiation. However, the split window technique requires parameterizations of surface variables such as air temperature and vapor pressure [21] and many parameters are assumed constant in space and time. Additionally, validation of estimates of solar radiation derived from remote sensing data are difficult because there are few observational constraints other than the remote sensing data used as input to the method itself.

We propose here a complimentary technique that integrates both ground-based and remote sensing observations to predict spatiotemporal patterns in downward solar radiation. The resulting method leverages the accuracy of ground-based pyranometers together with the spatiotemporal coverage afforded by remote sensing data. The method is specifically based on machine learning algorithms widely used in climatology and remote sensing [22–23]. Compared with traditional methods for estimating downward solar radiation, a machine learning approach holds several key advantages. A machine learning approach can (1) be used to identify those variables that are most powerful in describing spatiotemporal variation in downward solar radiation, (2) provide explicit mechanisms for quantifying uncertainties in predicted values of solar radiation, (3) leverage diverse kinds of remote sensing data including multispectral imagery and lidar-derived vegetation and elevation characteristics, (4) capture potentially non-linear relationships between independent and dependent variables, and (5) provide an assessment of model robustness.

The overarching goals for this study are to: (1) test the degree to which a machine learning approach using random forests can accurately develop predictive models of surface downwelling solar radiation using a combination of variables from remote sensing datasets, (2) understand and provide justification for the presence and prevalence of predictor variables used in the random forest model, (3) analyze the uncertainties in predictions of surface downward solar radiation, and (4) use the random forest model to extrapolate predictions to the scale of an entire watershed and assess the derived spatiotemporal patterns.

## 2. Methods

### 2.1 Research area

**Fig 1:**
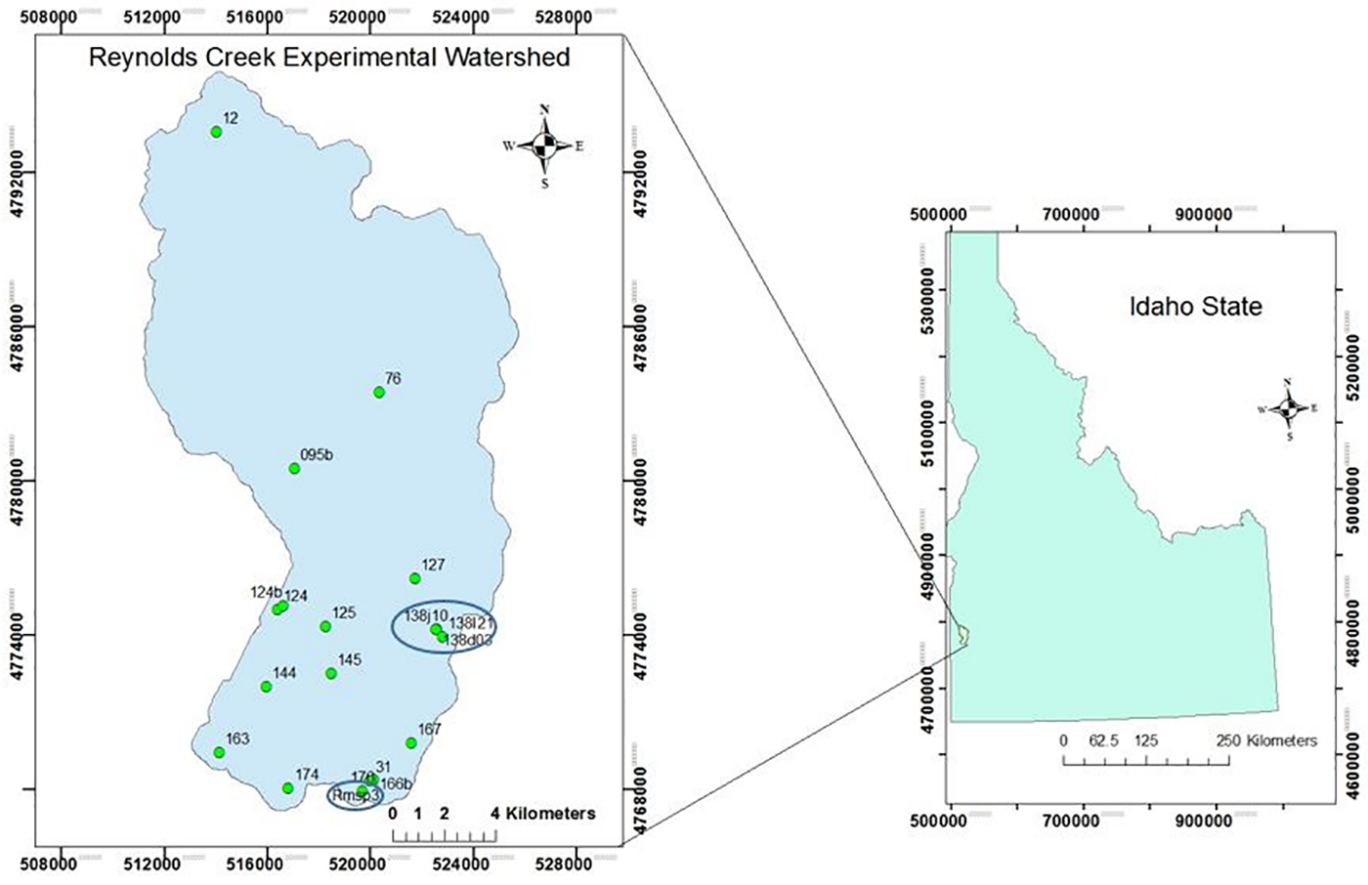
Reynolds Creek Experimental Watershed with 16 meteorological sites (used for model development and validation) and additional subsets used for validation (in circles).

Reynolds Creek Experimental Watershed (RCEW) is 239 km^2^, located in the rangelands of the Owyhee Mountains in southwestern Idaho, USA (Fig 1). The US Department of Agriculture’s Agricultural Research Service (ARS) established RCEW in 1960 as an experimental platform to understand and characterize impacts of rangeland management activities on hydrology, ecology, and geomorphology. Since its establishment RCEW has been the focal point of many studies focusing on terrestrial vegetation, soil science, hydrology, and hydroclimatology, and most recently as a Critical Zone Observatory (CZO). The primary drainage of the watershed, Reynolds Creek, flows primarily from south to north. Elevation in RCEW ranges from 1099 m at the outlet weir to approximately 2093 m at the southern end of the watershed [24]. The RCEW is a semi-arid ecosystem dominated by sagebrush-steppe in the lower elevations and large stands of coniferous and deciduous trees at higher elevations in the watershed. Mean annual precipitation varies greatly in both amount and phase in RCEW, with about 240 mm falling (primarily as rain) at lower elevations in the watershed and greater than 1100 mm falling (primarily as snow) at higher elevations [25; http://criticalzone.org/reynolds/about/]. The climate in RCEW is characterized by hot and dry summers and mild and wet winters. The vast majority of the precipitation falls in the period between December and March. Steeper hillslopes in the watershed tend to be associated with shallow and rocky soils while more gently graded hillslopes tend to be associated with deeper, loamy soils. RCEW possesses a rich long-term dataset of key climate variables including temperature, solar radiation, humidity, wind, precipitation, snow and stream flow that has been recorded since the 1960s [26].

### 2.2 Datasets

#### 2.2.1 Ground-based measurements of solar radiation

The ARS Northwest Watershed Research Center, which operates RCEW, maintains 16 meteorological stations (Fig 1) within the watershed associated with three subsets. Generally, these stations provide long-term precipitation, temperature, humidity and solar radiation data. Surface observations at these meteorological stations were initiated at different times in the past, with site 76 having the longest continually running monitoring period dating from 1964 [26]. Detailed information about these measurement sites is provided in Table 1. These 16 sites cover a wide range of elevations throughout the watershed, ranging from 1533 to 2169 m, and are associated with a variety of vegetation cover characteristics and hillslope aspects. Observed downward solar radiation from these sites constitute the surface observations used to develop our predictive models. In particular, we focus on the year 2007 since the instrumental record contains no temporal gaps in this year. At these 16 sites, downward solar radiation fluxes are measured with Eppley precision spectral pyranometers, which are sensitive to wavelength from 0.285 μm to 2.800 μm. In this study we are interested in predicting the solar flux throughout the watershed as a measure of total solar energy input to the terrestrial ecosystem. Therefore, at each site, we used the average of solar flux data for every day to represent daily solar flux data. The solar flux data is expressed in units of W/m^2^. Downward solar radiation and other historical hydroclimate data can be accessed through the NWRC website: ftp://ftp.nwrc.ars.usda.gov/.

**Table 1:** The location, elevation and the start dates for collecting downward solar radiation for the meteorological sites in RCEW, where 16 sites are used for training and validation, and three additional subsets used for validation.

**Site name**	**Latitude (m)**	**Longitude(m)**	**Elevation(m)**	**Start date**
**163**	514133.8	4769428.0	2169	11/29/99
**167**	521600.5	4769781.0	2002	12/08/99
**174**	516813.1	4768022.0	2074	09/18/01
**176**	519689.9	4767928.0	2093	02/24/83
**125**	518266.2	4774328.0	1508	06/26/02
**127**	521745.3	4776195.0	1649	12/05/84
**144**	515949.4	4771988.0	1814	09/20/01
**166b**	520140.5	4768361.0	2067	10/02/03
**076**	520365.3	4783423.0	1200	06/18/81
**095b**	517063.5	4780455.0	1533	05/14/03
**124**	516395.2	4774980.0	1804	07/01/02
**145**	518476.7	4772497.0	1585	10/02/01
**012**	514030.0	4793587.0	1575	06/29/00
**124b**	516620.7	4775132.0	1778	10/31/06
**031**	519976.3	4768322.6	1794	08/08/00
**138d03**	522592.5	4774215.0	1869	01/21/04
**Three additional subsets in RCEW used for validation**				
**Site name**	Latitude (m)	Longitude(m)	Elevation(m)	Start date
**Rmsp3 (located in same pixel as 176)**	519976.3	4768322.6	2056	10/27/98
**138j10 (located in same pixel as 138d03)**	522562.7	4774200.0	1894	07/17/03
**138I21 (located in same pixel as 138d03)**	522799.4	4773911.2	1999	07/16/03

#### 2.2.2 MODIS remote sensing products

Three products from the MODerate-resolution Imaging Spectroradiometer (MODIS) sensor on NASA’s Terra and Aqua satellites are used in this study: (1) MODIS Albedo (combined Terra and Aqua) product (MCD43B3, Version 5), (2) MODIS/Terra Land Surface Temperature and Emissivity (LST/E) product (MOD11A1, Version 4), and (3) MODIS/Terra Vegetation Indices product (MOD13A2, Version 5). Each of these products is available at a 1 km spatial resolution over land areas globally. MODIS products can be downloaded from the Land Processes Distributed Active Archive Center (LPDAAC) website (https://lpdaac.usgs.gov). The MCD43B3 product provides surface albedo information at 8 day intervals. Note only the eight-day MODIS albedo product was available during the timeframe of this study. The dataset combines observations from both the Terra and Aqua satellites and the retrieval algorithms developed for generating this product make use of MODIS spectral data at seven spectral bands (0.648 μm, 0.858 μm, 0.470 μm, 0.555 μm, 1.240 μm, 1.640 μm, and 2.130 μm) and three additional broad bands (0.3–0.7 μm, 0.7–5 μm, 0.3–5 μm) [27–29]. The black and white sky albedo represents directional hemispherical reflectance (at solar noon) and bihemispherical reflectance (under conditions of isotropic illumination), respectively. The data product provides surface anisotropy, black and white sky albedo, nadir (i.e., view-angle corrected) surface reflectance, and key quality control information, which is stored as the MCD43B2 product (https://lpdaac.usgs.gov/dataset_discovery/modis/modis_products_table/mcd43b2). The MCD43B3 has attained validation stage 3 (high quality validation). Overall, the high quality MODIS operational albedos are well less than 5% albedo at the validation sites and the low quality albedos are within 10% of the ground-based measurements (http://landval.gsfc.nasa.gov/ProductStatus.php?ProductID=MOD43), both of which are acceptable. We included this product in our model because of the contribution of albedos on downward solar radiation.

MOD11A1 is a Terra land surface temperature and emissivity product, which provides daily per-pixel temperature, emissivity, clear day coverage, and night/day coverage. This MODIS product is retrieved by a split-window algorithm and validated with in-situ measurements [30–31]. The quality assurance information can be found from http://www.icess.ucsb.edu/modis/LstUsrGuide/usrguide_1dtil.html#qa. The Terra land surface temperature and emissivity product was included in the model due to the role of clouds reflecting downward solar radiation.

The MOD13A2 product provides spatiotemporal coverage of vegetation conditions via several indices. The quality assurance information can be found from https://lpdaac.usgs.gov/dataset_discovery/modis/modis_products_table/mod13a2. Two particular vegetation indices of interest in this study include the normalized vegetation index (NDVI) and Enhanced Vegetation Index (EVI), available at 16 day temporal intervals. The blue, red and near-infrared reflectance, at 0.469 μm, 0.645 μm, and 0.858 μm, are also used. Note that the estimated downward solar radiation in this study corresponds to that below the vegetation canopy.

#### 2.2.3 Computation methods

To construct the daily data for the albedo and vegetation parameters for each pixel containing ground albedo data in 2007, the remote sensing time series from the MCD43B3 and MOD11A1 products are interpolated and smoothed. Twenty predictors from MCD43B3 and seven predictors from MOD13A2 are obtained from the interpolation and smoothing scheme, which is similar to the scheme used by Dozier et al [32]. There are several reasons for the interpolation and smoothing. First of all, interpolation techniques can prepare the daily predictors for the model inputs and also increase the dataset size. Second, these two MODIS products contain intermittent time gaps due to cloud or weather conditions whereas concurrent ground-based observations may still be available during the same time period. The interpolation and smoothing algorithm (implemented in Matlab, MatlabR2015, The MathWorks, Inc.) is applied to the data to fill the spatial-temporal data along the time series to estimate *f*(*t*). The smoothing spline for the function is as follows (http://www.mathworks.com/help/curvefit/smoothing-splines.html):

$$f(t)=p{\sum_{j=1}^{N}w(j)\left|\hat{f}(t_{j})-f(t_{j})\right|}^{2}+(1-p)\int_{t_{\text{min}}}^{t_{\text{max}}}\lambda (t){\left|D^{2}f(t)\right|}^{2}dt$$ (1)

*w*(*t*) is the weight vector, the default value in the error measure is ones (size (x)). The default value for *λ*(*t*) is 1. *D*^2^*f*(*t*) is the second derivative of the function *f*(*t*). P is the smoothing parameter, which varies between 0 and 1. If p = 0, *f*(*t*) is the least-squares straight line fit to the data. If p = 1, *f*(*t*) is the variation cubic spline interpolant. p is usually chosen around 1 / (1 + *h*^3^ / 6), with h the average spacing Δ*t* of the data sites. After interpolation, the daily data for the predictors are obtained.

The study domain is located entirely within row four and column nine of the MODIS Sinusoidal Tile Grid. The MODIS Reprojection Tools (MRT) were used to reproject these MODIS products from their native sinusoidal to a Universal Transverse Mercator (UTM) Zone 11 north projection.

### 2.3 Machine learning approach and validation methods

The machine learning algorithm random forest is an ensemble classifier that consists of many decision trees [33]. It uses bootstrap samples to construct multiple decision trees. Each decision tree is built on a random subset of the training samples. During the tree growing process, the best split of the data is determined through n randomly selected features. The samples that are not used in the bootstrap process are out-of–bag (OOB) samples. To evaluate the accuracy of the model, the classification error is estimated for tree samples that are not used for training the model. The average of these errors over the total number of trees is referred to the OOB error. In the study, 32 parameters extracted from the MODIS products are used to predict downward solar radiation (Table 2). Black sky albedo and white sky albedo at different spectral bands are used from MCD43B3, the land surface temperature and emissivity from MOD11A1, and vegetation parameters from MOD13A2. The RF model is developed here using Matlab's ClassificationBaggedEnsemble techniques. This algorithm trains learners from the data, combines a set of the trained models, and then aggregates the predictions based on the new data from the learners. This approach can also help in evaluating the importance of each indicator by estimating how many times the parameter is used in each model run, with parameters that are used more frequently considered relatively more important.

**Table 2:** The 32 parameters extracted from the MODIS products. Note: BSA is black sky albedo. WSA is white sky albedo.

MODIS Product	Data sets	Units
**MCD43B3**	Albedo_BSA_Band1	None
	Albedo_BSA_Band2	None
	Albedo_BSA_Band3	None
	Albedo_BSA_Band4	None
	Albedo_BSA_Band5	None
	Albedo_BSA_Band6	None
	Albedo_BSA_Band7	None
	Albedo_BSA_nir	None
	Albedo_BSA_shortwave	None
	Albedo_BSA_vis	None
	Albedo_WSA_Band1	None
	Albedo_WSA_Band2	None
	Albedo_WSA_Band3	None
	Albedo_WSA_Band4	None
	Albedo_WSA_Band5	None
	Albedo_WSA_Band6	None
	Albedo_WSA_Band7	None
	Albedo_WSA_nir	None
	Albedo_BSA_shortwave	None
	Albedo_BSA_vis	None
**MOD11A1**	Clear_day_cov	None
	Clear_night_cov	None
	Emissivity_31	None
	Emissivity_32	None
	Night_view_angl	Degrees
**MOD13A2**	1 km 16 day EVI	EVI
	1 km 16 day blue reflectance	Reflectance
	1 km 16 day MIR reflectance	Reflectance
	1 km 16 day NDVI	NDVI
	1 km 16 day NIR reflectance	Reflectance
	1 km 16 day red reflectance	Reflectance
	1 km 16 day sun zenith angle	Degree

For this research, we validate the models using three different approaches. In the first approach, the model is trained based on 12 of the 16 sites and which are selected from different site characteristics. After training the model, we use the model to predict the time series of downward solar radiation in 2007 for the remaining four sites which are not used for training. The four different combinations used for the unselected sites (Table 1) are 012, 076, 095b, 124; 124b, 125, 127, 128; 138d03, 144, 145, 163; and 174, 176, 166b, 167. The bias, root mean square error (RMSE), mean absolute error (MAE) and mean absolute percentage error (MAPE) are calculated using these sites. The second approach validates the model simulation result using data from subsets within the same MODIS pixel. Sites 138j10 and 138I21 are within the same pixel as site 138d03, and site 176 is within the same pixel as site Rmsp3. Therefore, we use sites138d03, 138I21 and 176 for independent validation. In the third validation approach, we develop an algorithm that is similar to a bootstrapping approach to randomly select 12 of the 16 sites used for training (a total of 1820 possible combinations ($C_{16}^{12}=1820$). Accordingly, we run the model 1820 times and calculate error metrics for the 12 site combinations. In the end, a total mean error for all of the model simulations is calculated.

We use different metrics to evaluate the agreement between model simulations and observed downward solar radiation data including RMSE, bias and MAE, and MAPE [34]. These metrics are calculated as follows:

$$RMSE=\sqrt{\frac{\sum_{i=1}^{n}{\left(X_{obs,i}-X_{pre,i}\right)}^{2}}{n}}$$ (2)

$$BIAS=\frac{1}{n}\sum_{i=1}^{n}\left(X_{obs,i}-X_{pre,i}\right)$$ (3)

$$MAE=\frac{1}{n}\sum_{i=1}^{n}\left|X_{obs,i}-X_{pre,i}\right|$$ (4)

$$MAPE=\frac{100}{n}\sum_{i=1}^{n}\left|\frac{X_{obs,i}-X_{pre,i}}{X_{obs,i}}\right|$$ (5)

Where *X*_*pre*,*i*_ is the predicted value at time t; *X*_*obs*,*i*_ is the observed value at time t; $\bar{X_{pre}}$ and $\bar{X_{obs}}$ are the average observed and predicted values at time t, respectively; and n is the number of observations.

## 3 Results

### 3.1 Interpolation of daily data for 32 predictors from MODIS products

**Fig 2:**
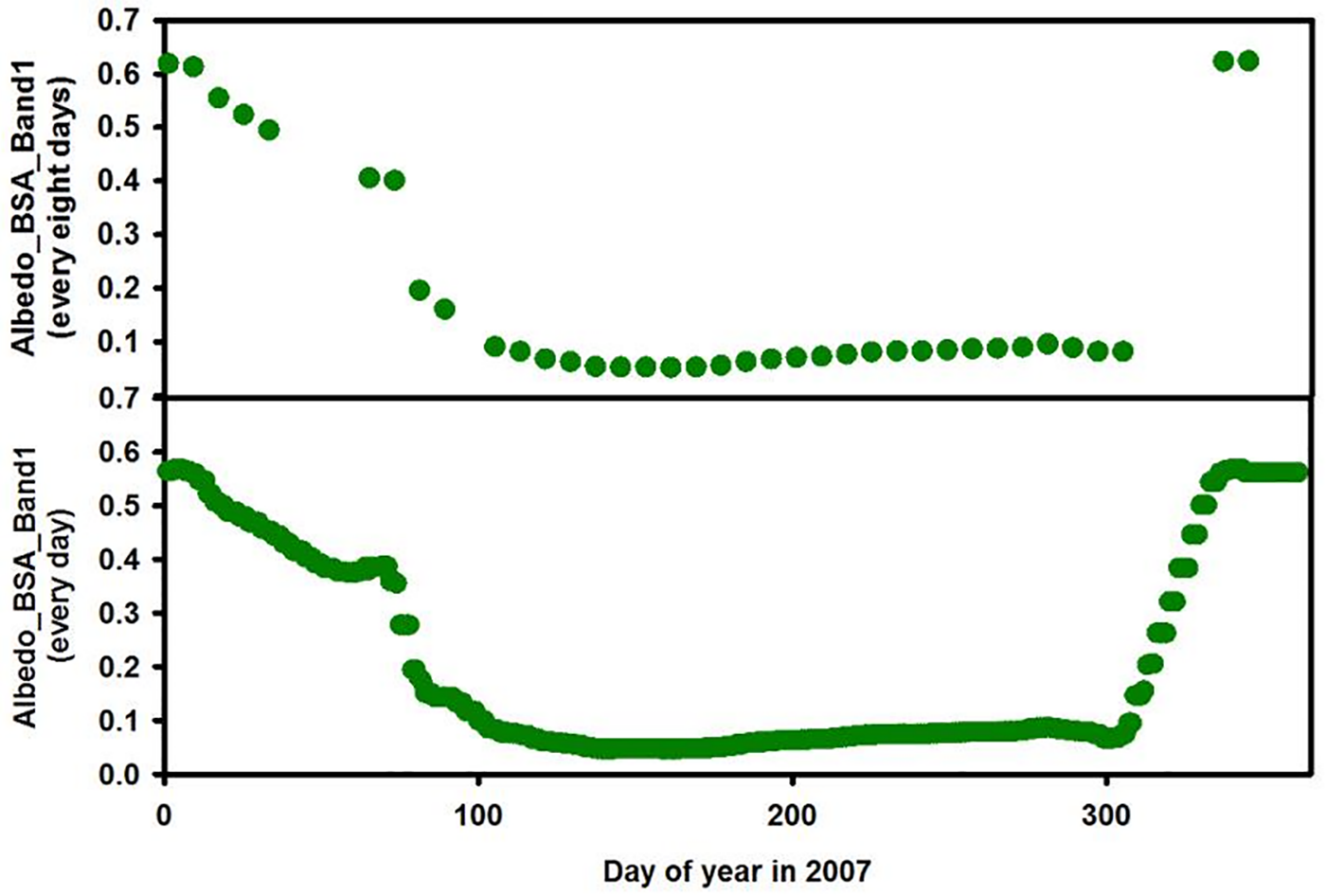
One example (site 176) showing the interpolation of daily data for Albedo_BSA_Band1 in 2007 from MCD43B3 which provides data every 8 days. The upper plot shows Albedo_BSA_Band 1 retrieved from MCD43B3 and the bottom plot shows daily interpolated Albedo_BSA_Band1 data.

The 32 predictors from MODIS (Table 1) are used to predict solar radiation. We use black sky albedo band 1 (BSA_Band1) at site 076 (elevation: 1200 m, Table 1) as an example to demonstrate the effects of the interpolation and smoothing algorithm. The upper plot in Fig 2 is black sky albedo with an eight-day interval and the bottom plot shows daily black sky albedo. Both plots suggest that albedo is changing seasonally with large fluctuations for BSA_Band1. In the first 90 days (late winter and early spring), there is a marked peak (0.61). Gradually the BSA_Band1 starts decreasing until around 0.2 at the 90^th^ day. After 90 days (late spring into summer), BSA_Band1 sharply decreases from 0.4 to 0.08 and remains low until around 300 DOY (winter). These phenomena are likely due to snow, dust, and/or plant phenology. In the early spring, the snow has not melted yet. Thus, the ground surface reflects more radiation and correspondingly, the albedo values are high. However, due to ablation of snow and increased vegetation growth, BSA_Band1 starts to decrease during late spring. During summer the BSA_Band1 values remain low until winter snow accumulation, at which point the values increase once again.

### 3.2 Model results and validation

**Fig 3:**
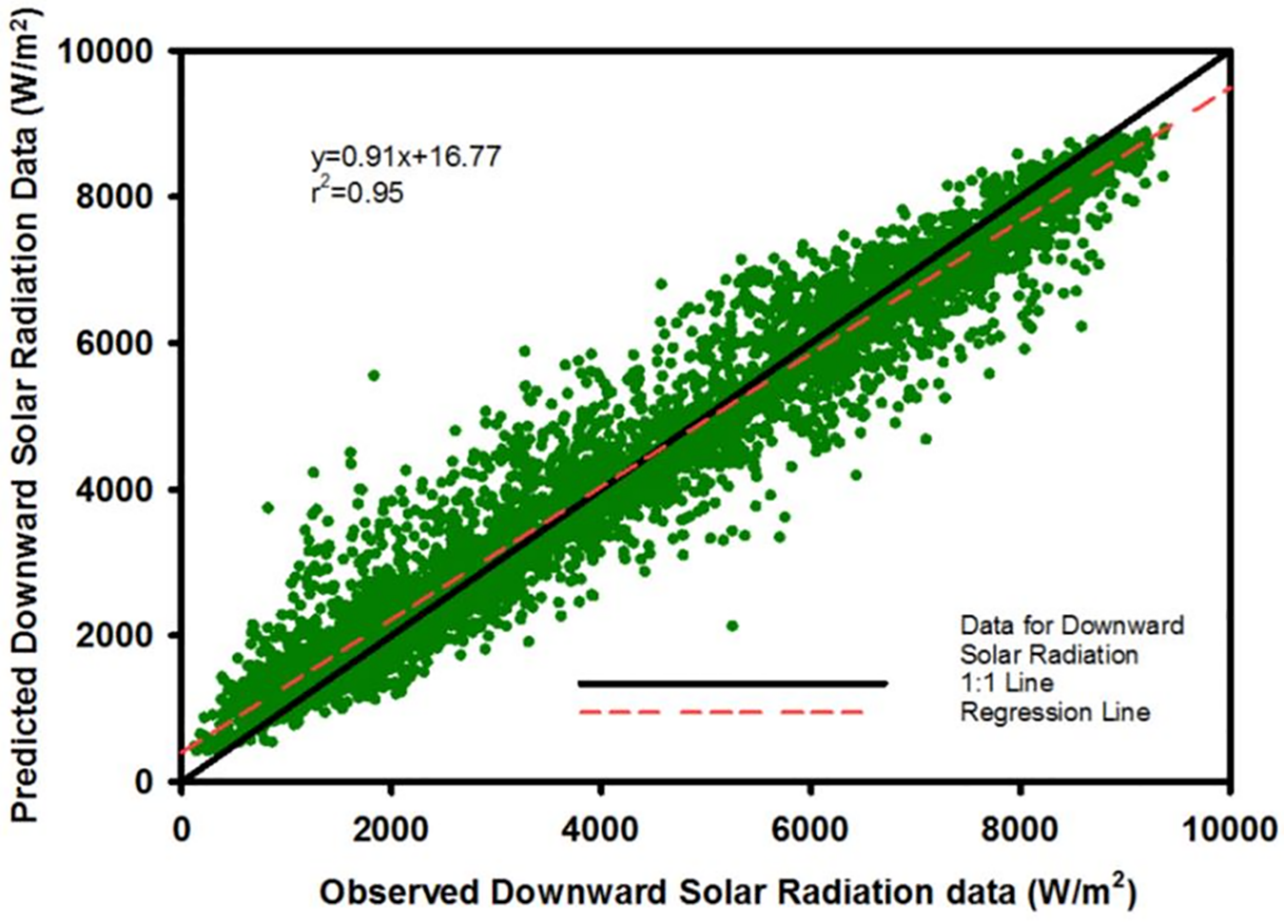
Model simulation results for the downward solar radiation based on random forest. The dashed red line represents the best fit and the solid black line shows 1:1 line. Note: R^2^ is for the regression line based on the observed and predicted downward solar radiation.

Daily downward solar radiation for the 16 sites are estimated by the RF model (Fig 3). Overall, the agreement between the model simulation and observation data is strong (r^2^ = 0.96; Fig 2). The mean simulated downward solar radiation (185.6 ± 93.5 W/m^2^) is close to the mean observed data (185.6 ± 100.3 W/m^2^). The model simulation and observation data range from 17.6 W/m^2^ to 372.4 W/m^2^, and from 5 W/m^2^ to 390.7 W/m^2^, respectively.

**Fig 4:**
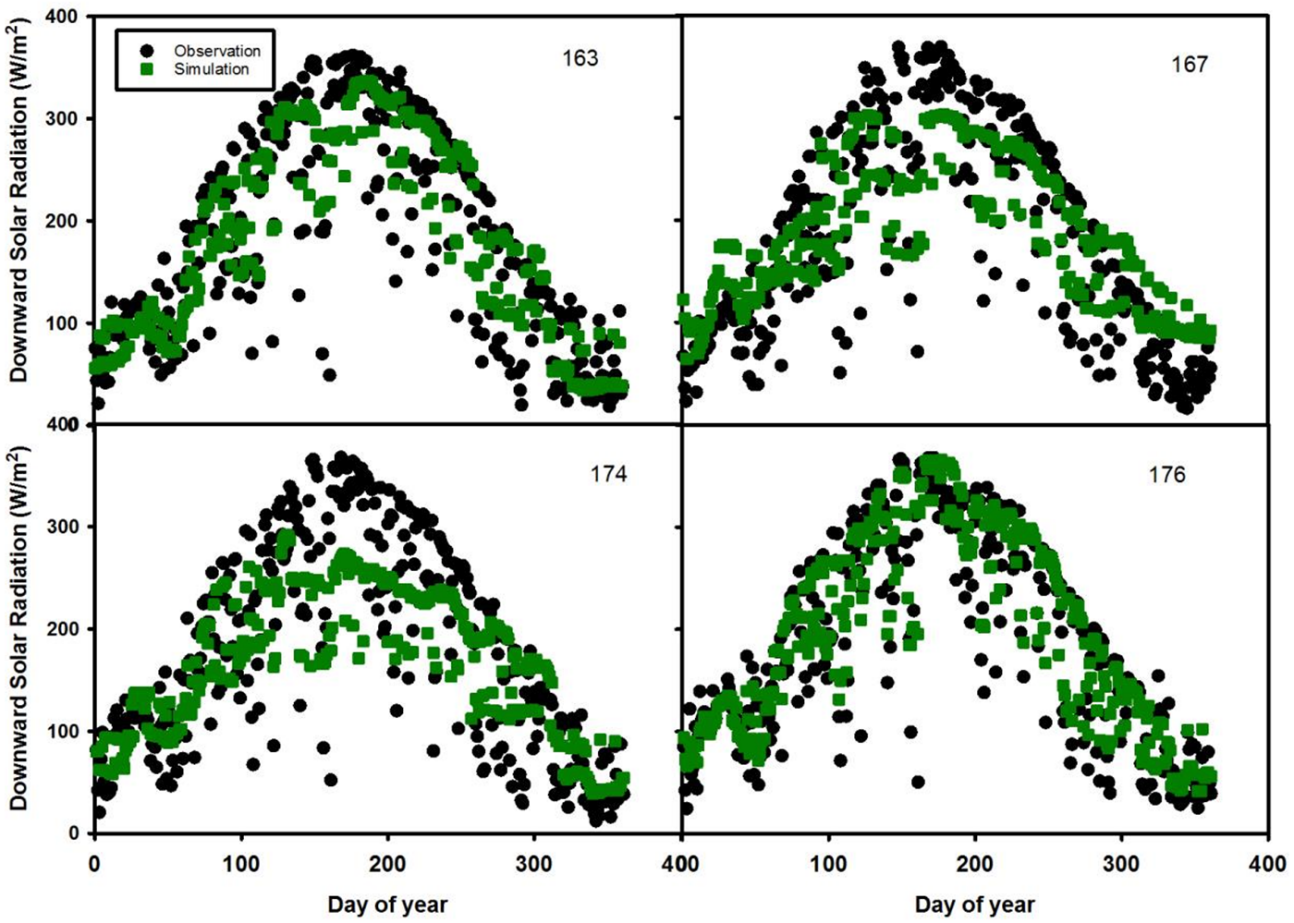
Time series of daily downward solar radiation in 2007 for test sites 163, 167, 174 and 176.

For the first validation approach, we present four combinations of predictions that are based on the remaining 12 sites (Table 3 and Fig 4). For combination 1, the model simulation results for sites 163, 167 and 176 capture the pattern of downward radiation although the prediction for site 174 underestimates the observation data. While site 176 agrees well with the observations, the peak periods in sites 163 and 167 do not. Site 176 has the lowest RMSE (25.5 W/m^2^) and site 174 has the largest RMSE (52.5 W/m^2^) among these four sites.

**Table 3:** The mean prediction and observation values, along with root mean square error (RMSE, W/m^2^), bias (W/m^2^), mean absolute error (MAE, W/m^2^) for the validation sites based on 2007 full year data.

Experiments	Sites	Mean_pre	Mean_obs	RMSE	Bias	MAE	MAPE (%)
**Combination 1**	163	181.78	184.10	39.96	-2.32	29.96	25.06
	167	187.00	184.084	50.38	2.92	41.17	41.23
	174	165.92	181.64	52.48	-15.72	41.64	31.52
	176	195.76	190.74	25.54	5.02	17.88	15.41
**Combination 2**	125	194.52	172.35	44.33	22.16	33.70	58.75
	127	150.03	175.71	69.80	-25.68	54.08	42.15
	144	190.50	184.62	38.91	5.88	29.02	27.02
	166b	190.58	196.53	28.20	-5.94	21.47	15.22
**Combination 3**	76	198.43	179.32	45.55	19.11	35.29	40.83
	095b	189.97	192.99	39.28	-3.02	29.62	23.38
	124	196.40	197.40	38.80	-0.99	29.08	21.82
	145	163.11	180.86	55.89	-17.759	44.78	41.07
**Combination4**	12	116.12	174.65	85.57	-58.53	69.40	41.34
	124b	193.43	197.40	36.73	-3.97	27.20	13.19
	31	173.12	196.53	62.73	-23.40	48.56	13.44
	138d03	190.74	190.74	57.09	-17.62	44.36	14.33

**Fig 5:**
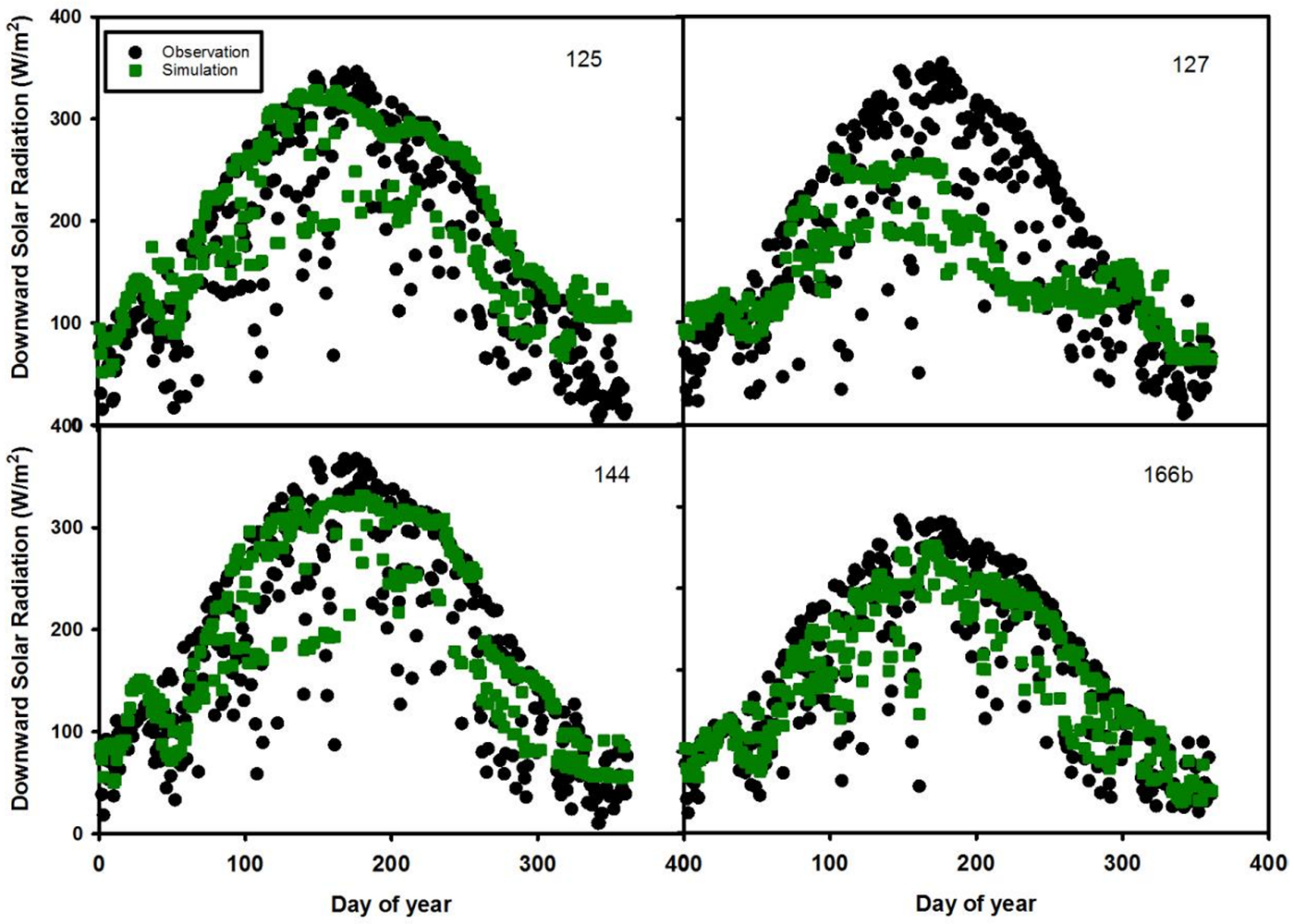
Time series of daily downward solar radiation in 2007 for test sites 125, 127, 144, and 166b.

In the second combination (site 125, 127, 144 and 166b (Table 3 and Fig 5)), sites 125, 144 and 166b agree well with the observation values but slightly underestimate the observation values during summer. The model fails to capture the pattern of downward solar radiation for site 127 from DOY 100 to 250.

**Fig 6:**
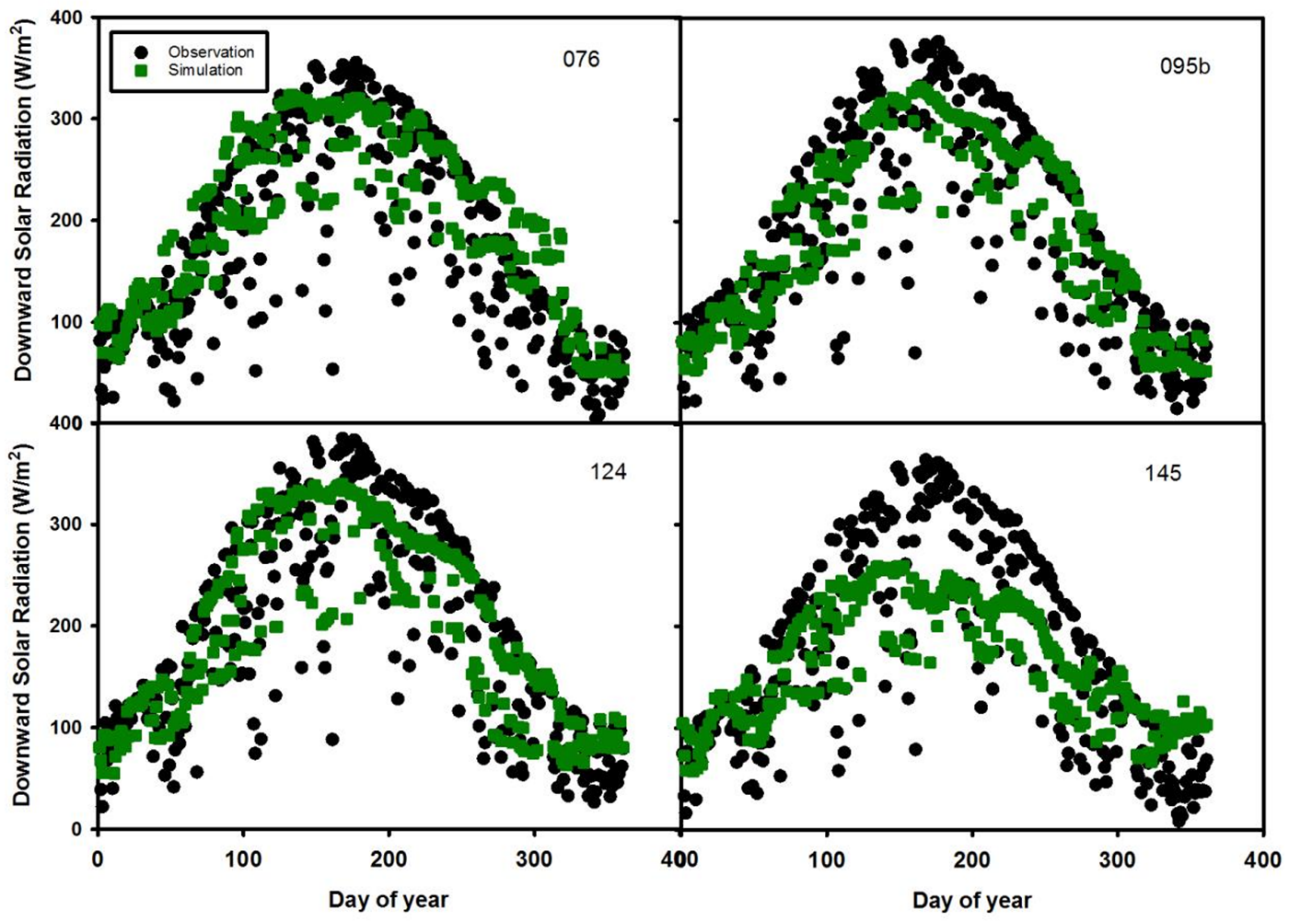
Time series of daily downward solar radiation in 2007 for test sites 076, 095b, 124, and 145.

In the third combination (sites 076, 095b, 124 and 145 (Table 3 and Fig 6)), sites 076, 095b and 124 match well with the observation data except slightly underestimating the observation data during summer. Site 145 (RMSE = 55.9 W/m^2^) doesn’t capture the pattern of downward solar radiation from DOY 90 to 220. The large difference between prediction and observation results is likely due to a large data gap for the predictors at site 145.

**Fig 7:**
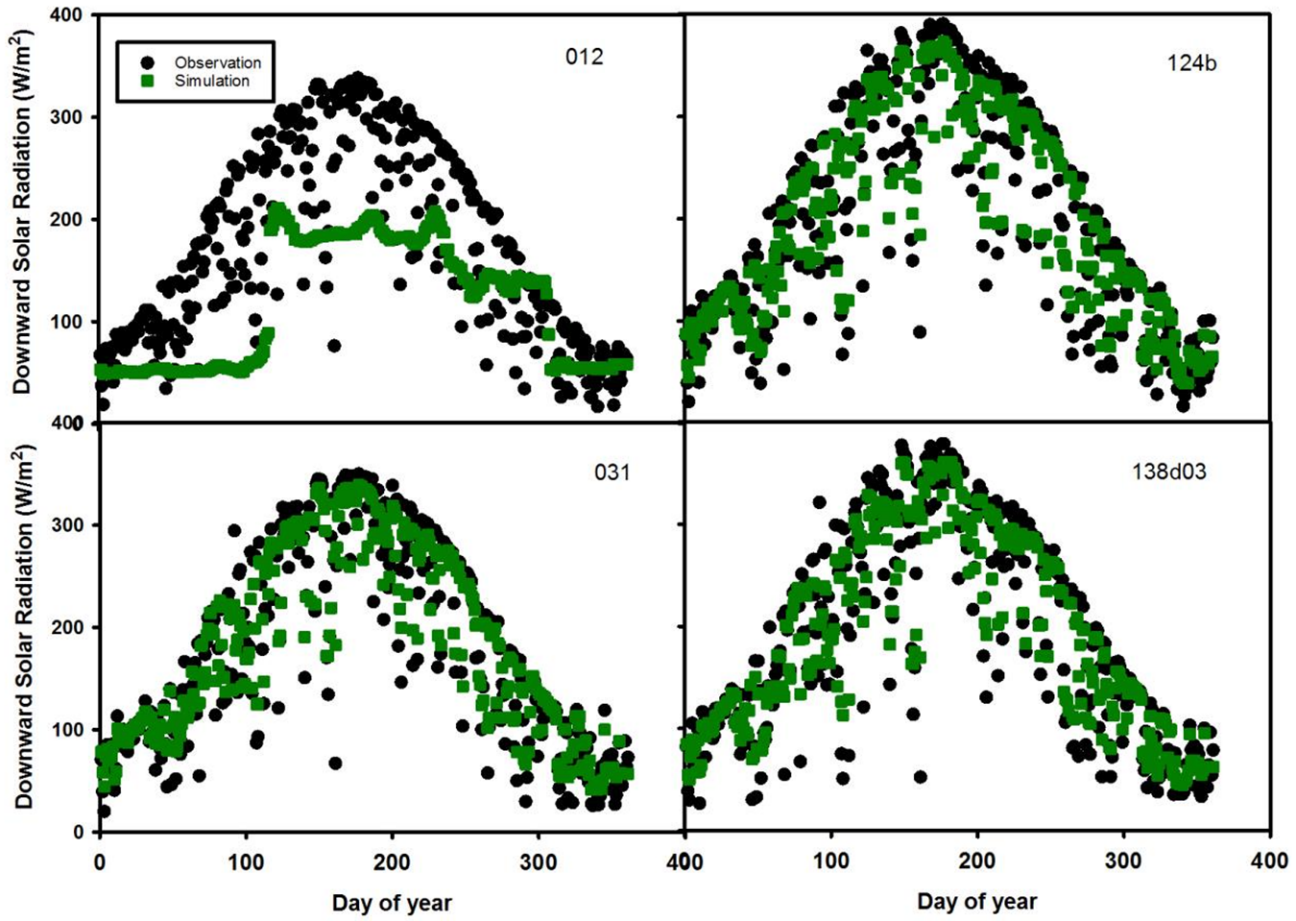
Time series of daily downward solar radiation in 2007 for test sites 012, 124b, 031 and 138d03.

The fourth combination uses sites 012, 124b, 031 and 138d03 (Table 3 and Fig 7). The model fails to predict site 012 (RMSE = 85.6 W/m^2^) but predicts the other three sites well: 124b (RMSE = 36.7 W/m^2^), 031 (RMSE = 62.7 W/m^2^) and 138d03 (RMSE = 57.1 W/m^2^).

**Fig 8:**
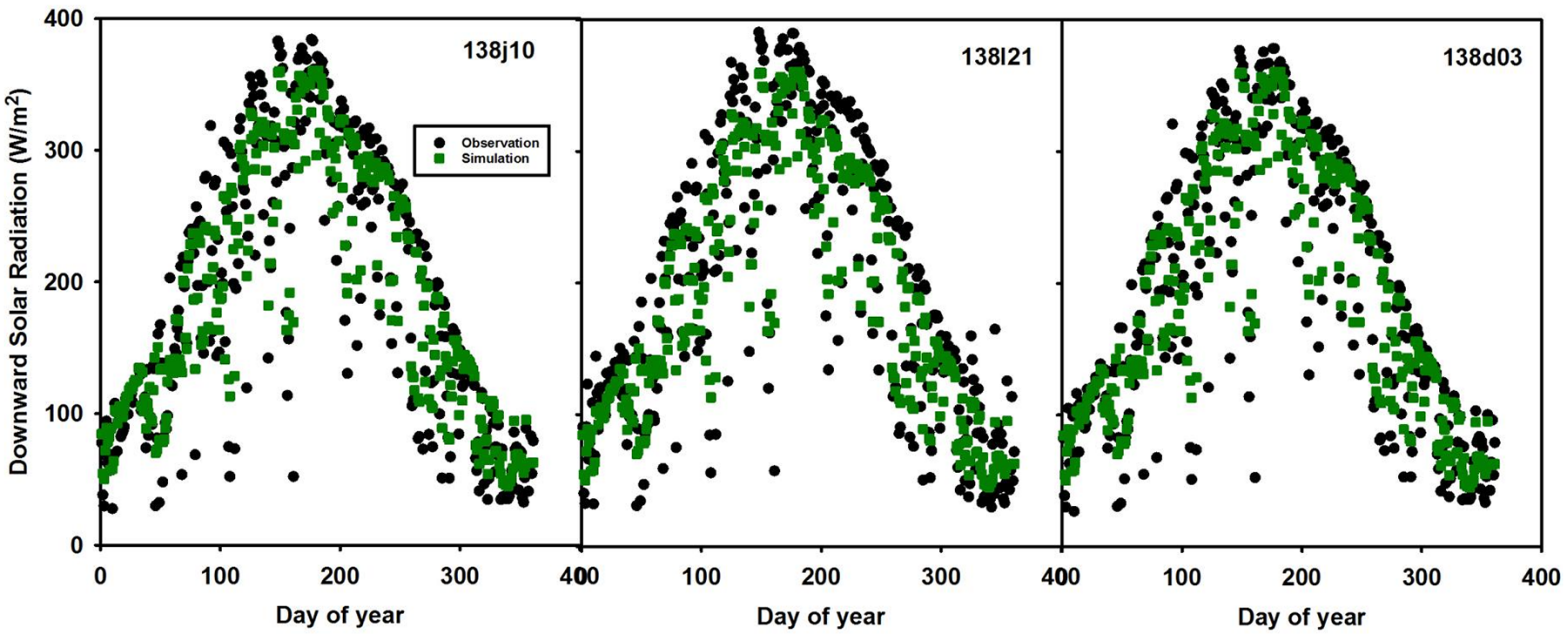
Validation sites 138j10 and 138I21 (located within same pixel as 138d03). The green dots are the model simulation data (identical within the same pixel) and the black dots are the observation data for the three different sites in the same pixel.

**Fig 9:**
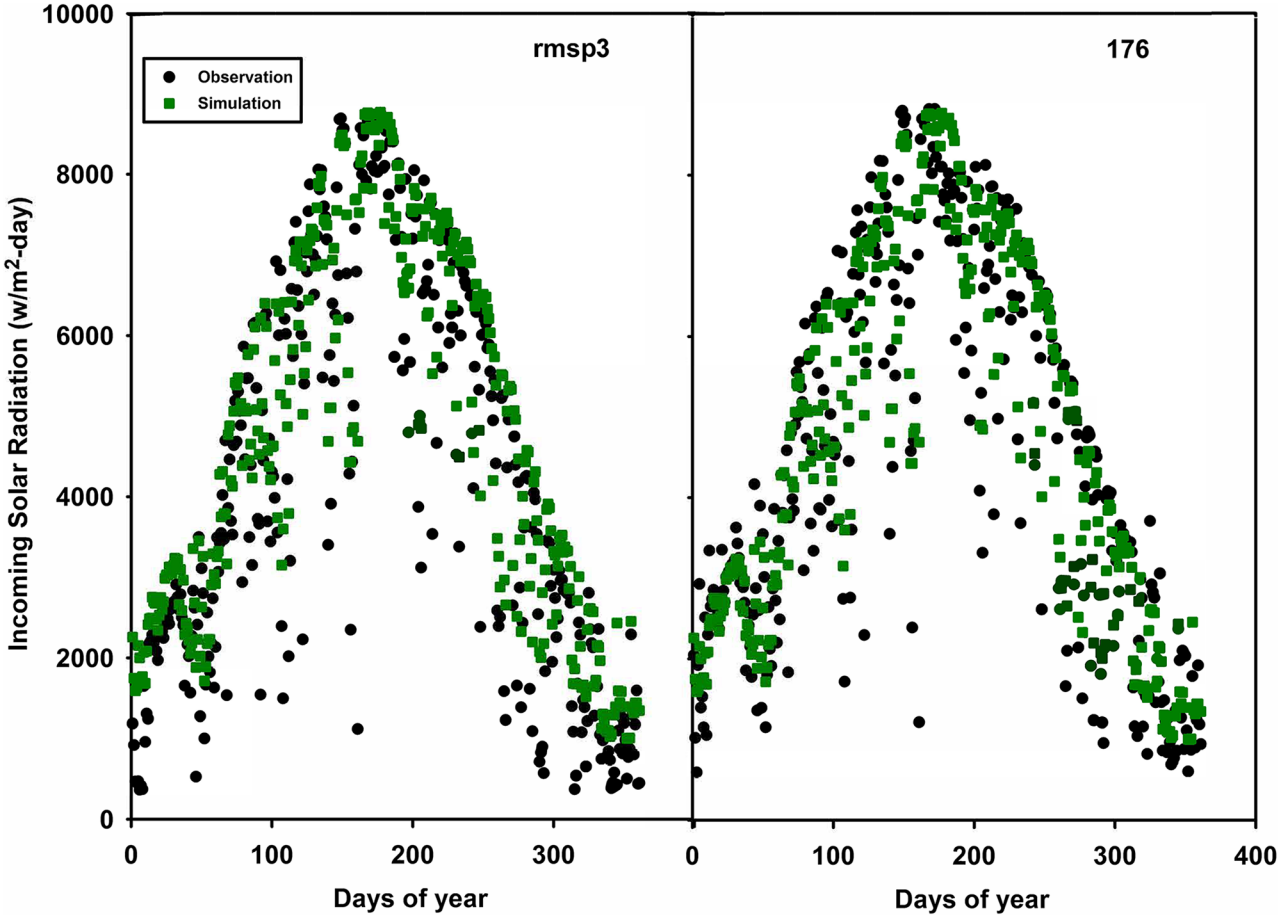
Validation site Rmsp3 (located within same pixel as 176). The green dots are the model simulation data (identical within the same pixel) and the black dots are the observation data for the two different sites in the same pixel.

Using our second validation approach, we used sites 138j10 and 138I21 as subsets of site 138d03 (Figs 8 and 9). Site 138j10 matches better (RMSE = 16.0 W/m^2^) than site 138I21 (RMSE = 29.0 W/m^2^); however overall the prediction result captures the pattern for site 138I21. There are differences among the simulated and observed within the same pixel due to sensor sensitivities caused by weather conditions such as cloudy days when the sensor cannot fully capture the radiation. This possibly explains the sharp fluctuations for the observation data during summer. Similarly, sites rmsp3 and 176 fall within the same MODIS pixel in RCEW. RMSE for site rmsp3 is relatively smaller than site 176.

**Fig 10:**
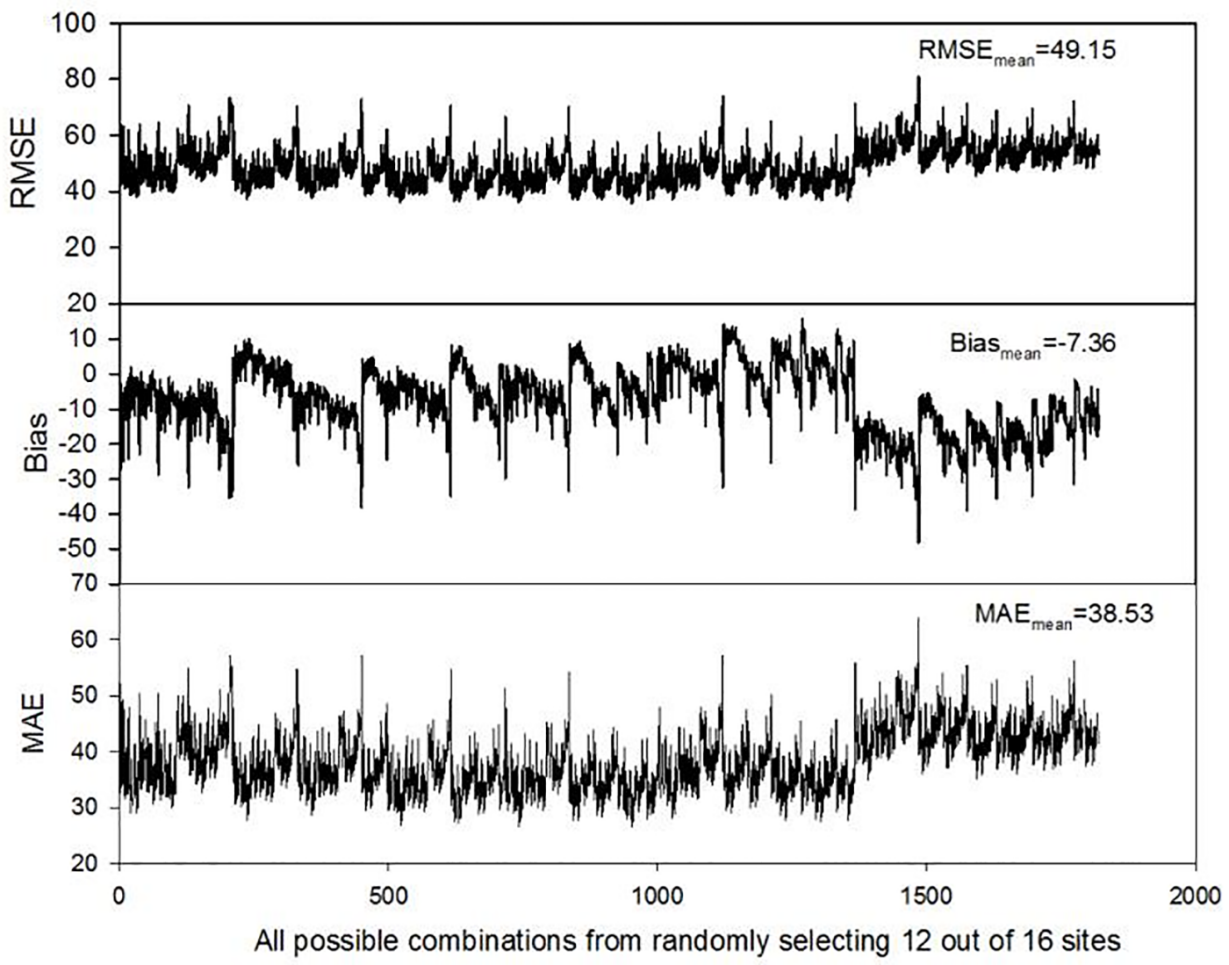
RMSE, Bias, MAE for all possible combinations based on RF model runs.

In the third validation approach, we run all possible combinations (n = 1820) using the model and evaluate the errors for each combination (Fig 10). The RMSE ranges from 37.2 W/m^2^ to 81.3 W/m^2^; bias ranges from -48.3 W/m^2^ to 15.7 W/m^2^ and MAE ranges from 26.6 W/m^2^ to 63.8 W/m^2^. These relatively high RMSE sites (peak values) are coincidentally the same sites with high bias and MAE values.

### 3.3 Distributed downward solar radiation for RCEW

**Fig 11:**
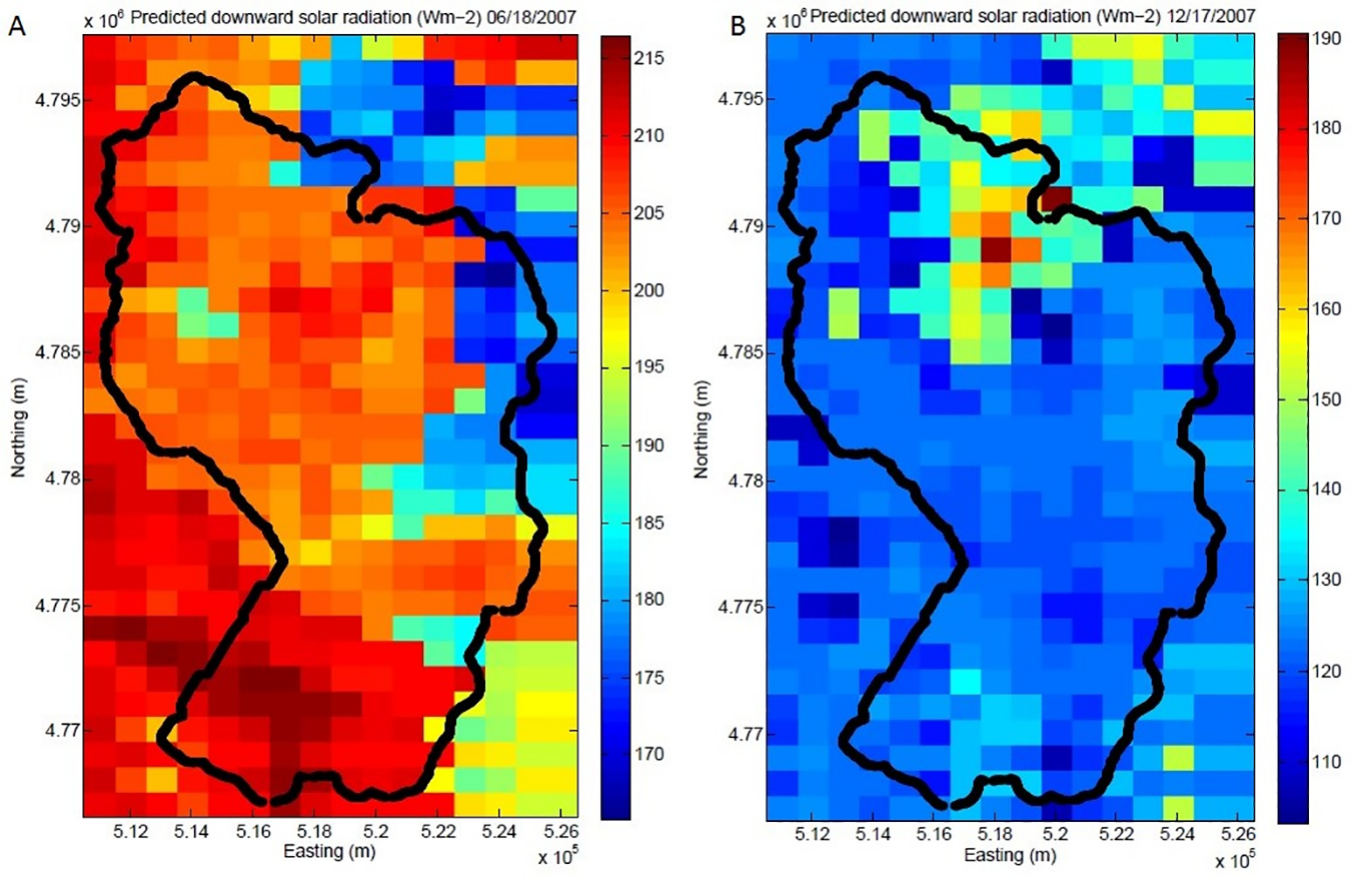
The spatiotemporal pattern of downward solar radiation (W/m^2^/day) for RCEW on June, 18^th^ (A) and on December, 17^th^ (B) in 2007.

We use two representative days, June 18^th^, 2007, and December 17^th^, 2007, as examples of the spatial distribution of solar radiation in RCEW. The downward solar radiation is obtained from the model based on the 16 sites associated with the 32 predictors for all pixels in the watershed. In June (Fig 11A), the downward solar radiation for the west side of the watershed is higher than that of the east side, decreasing from southwest to northeast and from 150 W/m^2^/day to 220 W/m^2^/day. The values range from 100 W/m^2^/day to 190 W/m^2^/day for December 17^th^ (Fig 11B), which are expectedly lower than the summer downward solar radiation.

### 3.4 Variable importance

**Fig 12:**
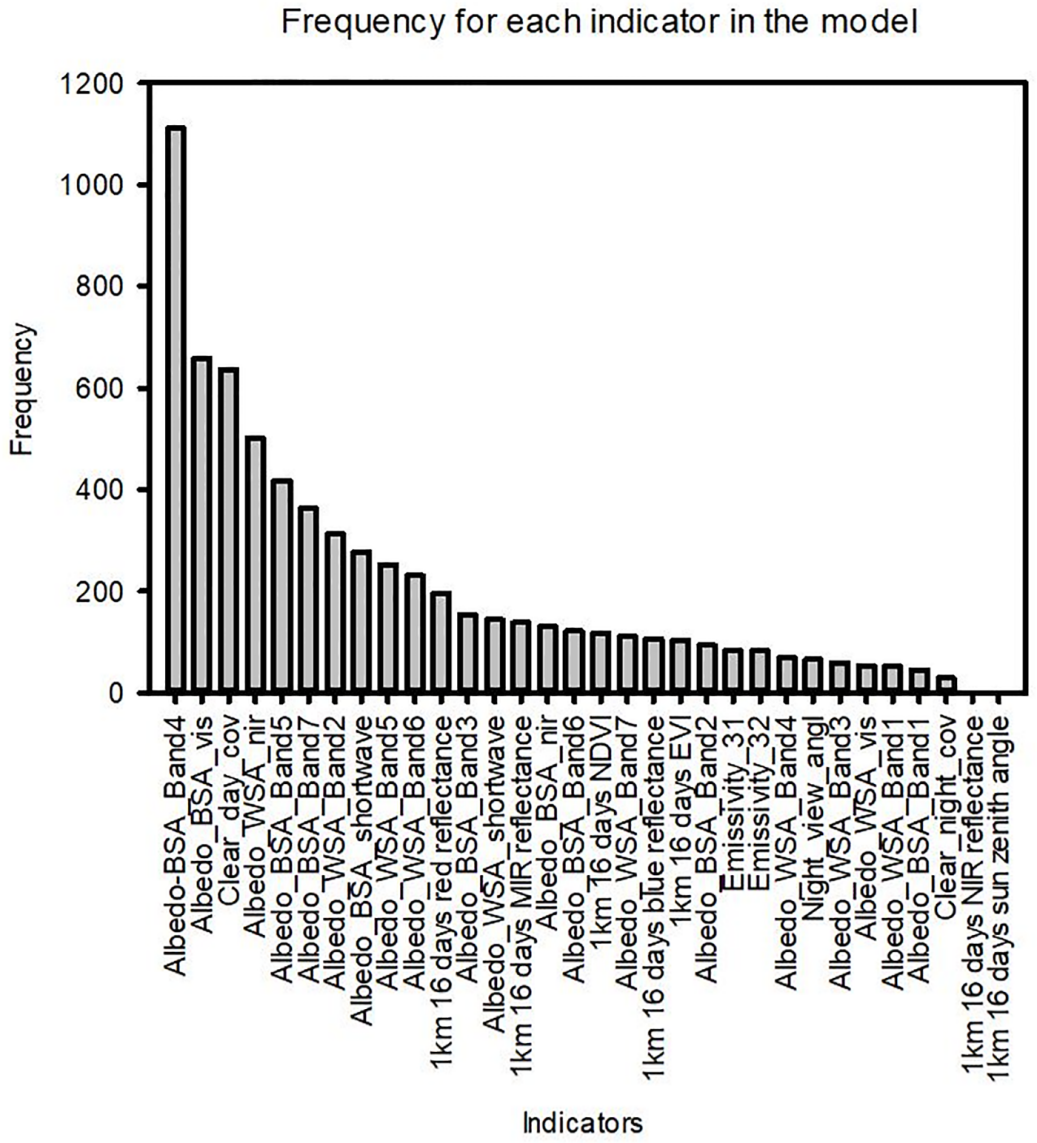
The important predictors used in the model based on all 16 sites.

We identified the frequency of the predictors used in the RF model to determine the most influential predictors for solar radiation (Fig 12). Generally, variables from the albedo product (MCD43B3) exhibit greater importance than those from the vegetation product (MOD13A2 and MOD11A1). Specifically, the three most important predictors belong to the albedo product, including the Albedo_BSA_Band 4 (0.470 μm) which was used most frequently (1110 times), the Albedo_BSA_vis (658 times) and the Clear_day_cov (636 times). These bands are followed by Albedo_WSA_nir (501 times), Albedo_BSA_Band 5 (0.555 μm, 417 times), Albedo_BSA_Band 7 (1.640 μm, 363 times) and Albedo_WSA_Band2 (0.858 μm, 312 times) (Fig 12).

## 4 Discussion

### 4.1 Evaluation for the model performance and comparisons for solar flux between June 18^th^ and December 17^th^, 2007

The RF model developed here effectively predicts downward solar radiation for DOY 1–365 for 2007 and explains 96% of the variance (Fig 3). We used three different approaches to validate the model output, all of which support our conclusion that solar radiation is reasonably predicted with MODIS variables yet nuances in data availability and seasonality are important to consider. Most sites show strong agreement between model simulation and observation data, with the exception of several sites with data gaps (Fig 2). The bootstrap approach demonstrates that the model underestimates summer values for all simulations. There are two possible reasons for this. First, the atmospheric dynamics are more active in summer and can cause changes in the atmospheric profile, affecting downward solar radiation. Furthermore, the 8- and 16-day intervals of the MODIS data may not provide sufficient temporal resolution in regards to the summer atmospheric dynamics. Second, the use of the interpolation and smoothing method to obtain daily predictors from MODIS products likely added uncertainty in the model and affected the model simulation for peak periods of downward solar radiation.

The three validation approaches have their own advantages and disadvantages. The simple regression analysis in the first approach doesn’t consider the spatial or temporal resolution. Further, it is challenging to define the location or time of outliers from Fig 3. The second validation approach (comparing sites within the same pixel), results in different model errors for each site (Figs 8 and 9). We expect differences in these results because of the 1 km pixel size and the complexity of the watershed. However, these differences between sites (within the same pixel) indicates the importance of spatial heterogeneity and points towards the need for potentially finer resolution modeling. Alternatively, the mean error of all the subsets within a pixel could be considered. The third validation approach minimizes human bias in selecting sites for training and validation. This objective assessment of the model errors may be the most representative of the overall uncertainty.

The spatiotemporal patterns of solar radiation in RCEW for June 18^th^ and December 17^th^, 2007, are useful to explore. During the summer period, downward solar radiation generally increases with elevation; however, this pattern is not consistent during the winter. The downward solar radiation decreases with increasing elevation possibly due to snow and cloud cover in the winter. The areas with snow or cloud cover are likely to have higher albedos which will reflect more downward solar radiation and thus the land surface will receive less solar radiation. The agricultural areas in RCEW (right plot, Fig 11) have the highest solar radiation in the winter period. These areas are also situated in low elevation and low topographic relief areas.

This approach has the potential to be applied to other regions in order to provide detailed and long-term downward solar radiation datasets for the ecosystem modeling community. While ground-based measurements of downward solar radiation are difficult to obtain, especially in complex terrain, this satellite-based approach can be used for estimating solar radiation across space and time.

### 4.2 Important predictors for downward solar radiation and model uncertainty analysis

The three most important predictors of solar radiation identified by the model are black sky albedo band 4, the black sky albedo (visible band) and clear day coverage. Both black sky albedo and white sky albedo are reflectance at a particular solar zenith angle. Numerous factors that affect albedo include the phenological cycle (agricultural green-up/harvesting), meteorological parameters (soil wetness or snow patterns), and climatological trends (desertification, and vegetation cover changes). As mentioned earlier, the albedo product has an 8-day time interval. If agricultural green-up/harvesting occurs during this 8-day period interval, the albedo is likely to introduce uncertainty to the estimation of solar radiation. Wet and melting snow are less than 0.60 and fresh snow albedo are more than 0.85 based on Zhang’s study [35] by comparing the impacts of these two types of snow. Fresh snow reflected more solar energy and reduced the absorbed solar energy than the wet and melting snow. Wildfire is also prevalent in RCEW and similar areas in summer and early fall, resulting in bare ground that reflects more solar energy and increase surface albedo.

The uncertainty of estimating downward solar radiation is also attributed to external errors from MODIS retrieval algorithms such as the kernel-based bidirectional reflectance distribution function (BRDF) model used by the atmosphere products [28]. In addition, errors may arise in the atmospheric correction process which estimates scattering and absorption attributed to aerosol optical depth and aerosol type [36]. Aerosol depth is relatively more accurate than aerosol type and properties 37]. Another possible factor that affects model uncertainty is the gap-filling methods used for missing data. Existing gap-filling methods include linear/nonlinear spatial interpolation, kriging etc [37]. In June and July (Figs 4–7), observed solar radiation is underestimated for several sites due to two reasons. The first reason is likely due to bias in the ground based measurements of solar radiation from climatic conditions [24]. The second reason is likely due to the MODIS products used in the random forest model. Based on Liang’s [38] study, MODIS-observed albedo tended to underestimate albedo in comparison from the ground based measurements, due to the retrieval algorithm.

While we analyze several types of errors that contribute to the estimation of solar radiation flux, more quantitative information is needed to understand the relative importance of these errors on the total uncertainty budget. Future work should focus on quantifying these errors and how scale affects solar radiation.

## 5 Conclusions

The results of this study indicate that combining ground-based and remotely sensed data can be used to quantify spatiotemporal patterns of solar radiation in a semi-arid ecosystem. We demonstrate that the RF model can be effectively used with predictors from MODIS products to predict downward solar radiation. With additional error analysis, long-term daily datasets of downward solar radiation using remotely sensed data and ground-based data may be readily available using our methods.
